# Unfolded protein response triggers differential apoptotic mechanisms in ovaries and early embryos exposed to maternal type 1 diabetes

**DOI:** 10.1038/s41598-021-92093-3

**Published:** 2021-06-17

**Authors:** Aslı Okan, Necdet Demir, Berna Sozen

**Affiliations:** 1 Department of Histology and Embryology, School of Medicine, Akdeniz University, Antalya, 07070 Turkey; 2 Department of Genetics, Yale School of Medicine, Yale University, New Haven, CT 06510 USA

**Keywords:** Infertility, Metabolic disorders, Reproductive biology

## Abstract

Diabetes mellitus (DM) has profound effects on the female mammalian reproductive system, and early embryonic development, reducing female reproductive outcomes and inducing developmental programming in utero. However, the underlying cellular and molecular mechanisms remain poorly defined. Accumulating evidence implicates endoplasmic reticulum (ER)-stress with maternal DM associated pathophysiology. Yet the direct pathologies and causal events leading to ovarian dysfunction and altered early embryonic development have not been determined. Here, using an in vivo mouse model of Type 1 DM and in vitro hyperglycaemia-exposure, we demonstrate the activation of ER-stress within adult ovarian tissue and pre-implantation embryos. In diabetic ovaries, we show that the unfolded protein response (UPR) triggers an apoptotic cascade by the co-activation of Caspase 12 and Cleaved Caspase 3 transducers. Whereas DM-exposed early embryos display differential ER-associated responses; by activating Chop in within embryonic precursors and Caspase 12 within placental precursors. Our results offer new insights for understanding the pathological effects of DM on mammalian ovarian function and early embryo development, providing new evidence of its mechanistic link with ER-stress in mice.

## Introduction

Studies over the past few decades have highlighted how metabolic disorders can cause female reproductive dysfunction and alter developmental programming in offspring^1,2^. DM is a consequence of complex metabolic disorders characterised by hyperglycaemia arising from a lack of insulin secretion or action^3^. Reduced insulin secretion and/or function results in disrupted carbohydrate metabolism within the adult body extending to almost every tissue and organ, and thus is associated with long-term organ damage, dysfunction and ultimately failure^3^. In particular, DM has been shown to adversely affect female reproductive function, including delayed oocyte maturation, increased granulosa/cumulus cell apoptosis, decreased ovarian reserve and impaired cellular metabolism^4–6^. In 2017, the International Diabetes Federation (IDF) reported that approximately 199 million women in all around the world live with diabetes, predicted to almost double by the year 2040^7^.

In addition, DM also alters developmental programming in the offspring^8^. Studies spanning more than two decades have demonstrated a crucial window in early embryonic development with high sensitivity to signals from the mother’s reproductive tract^1,9,10^. During this window, around the time of conception and during the first zygotic divisions, the embryo is surrounded by the maternal oviductal fluid generating the nutritional and metabolic microcosm. This exposed microenvironment can influence the molecular pathways directing self-renewal, gene expression, intracellular stress mediators and survival in early embryos^1,9–12^. Cellular responses to the maternal environment are inherited through subsequent cell cycles, leading to a rewiring of the entire developmental program^9^.

**Figure 1:**
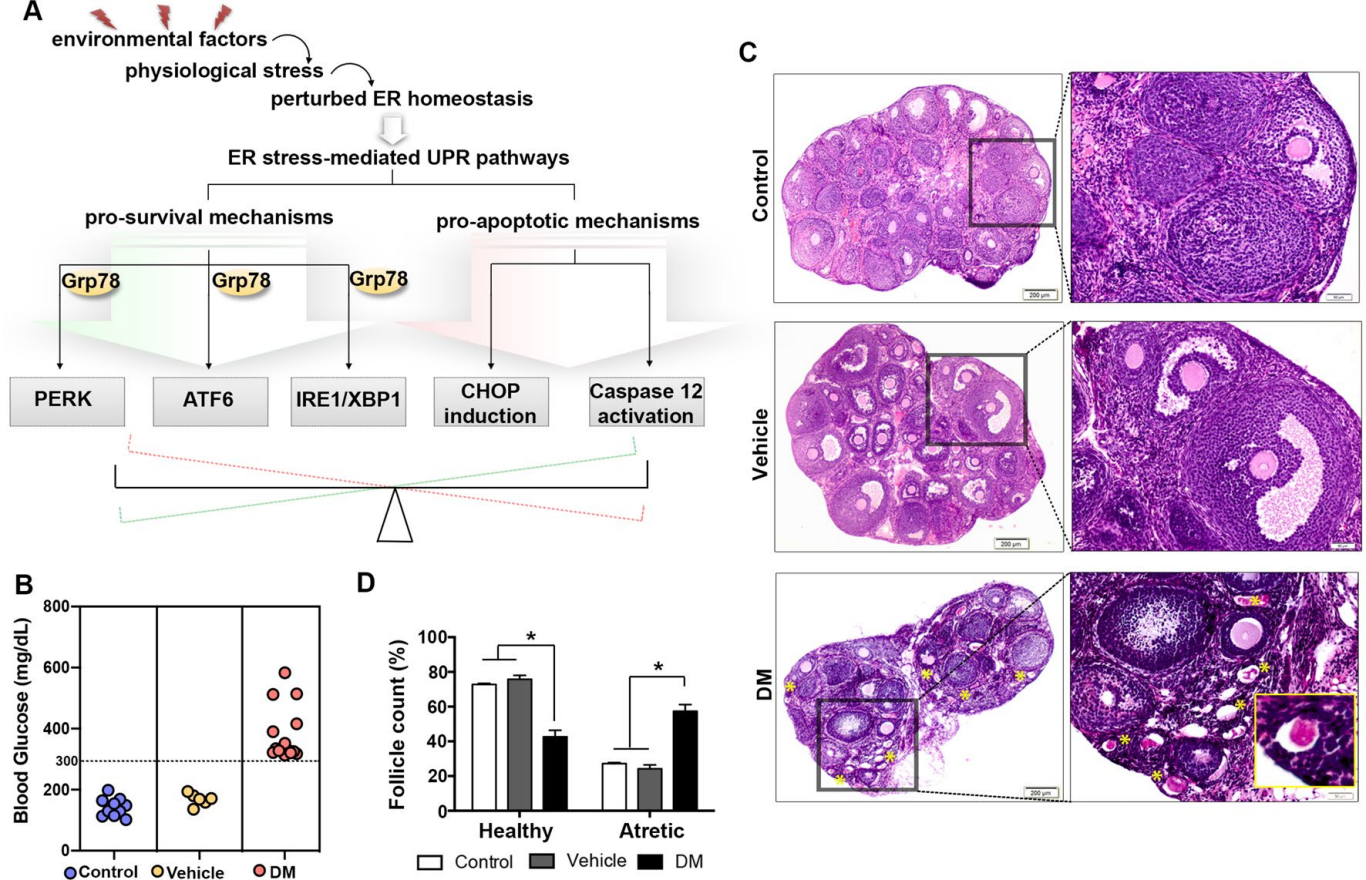
Histomorphometric evaluation of ovaries shows DM-induced follicular atresia. **(A)** Illustration depicts intracellular ER stress-mediated UPR pathways: pro-survival and pro-apoptotic mechanisms. (**B)** Blood glucose level measurement of experimental groups. Mice with glucose level ≥ 300 mg/dL were considered as DM. Control, n = 10; Vehicle, n = 6; and STZ-induced DM, n = 15. (**C)** Representative ovarian tissue micrographs of control, vehicle and DM groups. Images on the left show low magnification micrographs, boxed area shown on the right with a higher magnification. Yellow asterisks indicate atretic follicles observed in DM group. Inset shows a representative atretic follicle with eosinophilic stained degenerated oocyte in DM group. Scale bars = 200 μm for upper panels, 50 μm for lower panels. (**D)** Follicular count of ovaries from control, vehicle and DM groups shows that the percentage of healthy follicle number is significantly lower; the percentage of atretic follicle number was significantly higher in DM-exposed ovaries. Two-way ANOVA followed by TUKEY’s multiple comparison test, *P < 0.05 (n = 3 per group). Columns are means ± s.e.m.

Environmental factors leading to different physiological stresses and/or harmful metabolites perturb ER homeostasis and cause ER dysfunction in the cell. These then lead to accumulation and aggregation of unfolded or misfolded proteins in the ER lumen, termed *ER stress*^13,14^*.* To re-establish ER homeostasis and functionality, the unfolded protein response (UPR) is activated autonomously^13,14^. Under physiological conditions, binding of Glucose-regulated protein 78 (Grp78), the master UPR regulator, to the luminal domain of ER stress transducers inactivates them. Activation of the UPR starts with separation of Grp78 from the three ER stress transducers; protein kinase RNA-like ER kinase (Perk)^15^, activating transcription factor 6 (Atf6)^16^ and inositol requiring enzyme-1/X-box-binding protein (Ire1/Xbp1)^17^, to ensure the cell survival^18^. (Fig. 1A). However, if ER stress is severe and prolonged, UPR can switch from a pro-survival to a pro-apoptotic mechanism through the C/EBP homologous protein (Chop) or Caspase 12 transducers^19^, triggering cell death (Fig. 1A).

Increasing evidence implicate UPR and ER stress in the pathogenesis of DM^20,21^. While pathologies of ovarian function associated with local hyperglycaemic environment are well established^4,5^, the specific role of ovarian ER stress as a cause, has not yet been reported in type 1 DM. Moreover, while the adverse effects of maternal DM on embryo development have been long known^22,23^, whether hyperglycaemic maternal microenvironment caused by type 1 DM has impacts on early embryonic development via ER stress and UPR is unknown. This necessitates a comprehensive understanding of mechanisms underlying the adverse pathologies observed in women of reproductive age and in pregnancies with type 1 DM.

Here we explored the consequences of DM by analysing the activation of pro-survival and pro-apoptotic mechanisms of UPR on adult ovarian tissue and, during early mammalian embryo development. To accomplish this aim, we generated an in vivo mouse model of maternal type 1 DM induced by streptozotocin (STZ) injections^24,25^ or utilised in vitro environmental exposure to high glucose concentrations. Our results show that ER stress induced by type 1 DM triggers apoptotic UPR signalling by the sequential co-activation of Caspase 12 and Caspase 3 in the ovarian tissue. In contrast, pre-implantation embryos show high susceptibility to various ER-associated cellular degradation pathways. Altogether, this research contributes a new insight into the pathological effects of DM on ovarian physiology, and, provides further understanding towards developmental programming in offspring that are exposed to maternal DM.

## Results

### Insufficient activation of pro-survival UPR in response to ER stress in adult ovaries with DM

In order to investigate the consequences of DM related stimulation of ER stress on ovaries, we induced Type 1 DM by intraperitoneal injection of STZ^24,25^. We first confirmed that all mice treated with STZ became diabetic (Fig. 1B) and then evaluated ovarian histomorphology in each group (Fig. 1C). We identified that, after exposure to DM, a significant number of ovarian follicles undergo atresia as marked by characteristic strong eosinophilic staining in degenerated oocytes (Fig. 1C,D). Thus, our first observation indicates that cell death is triggered in adult ovaries under DM. We next investigated whether this was caused by the initiation of ER stress-related mechanisms.

**Figure 2:**
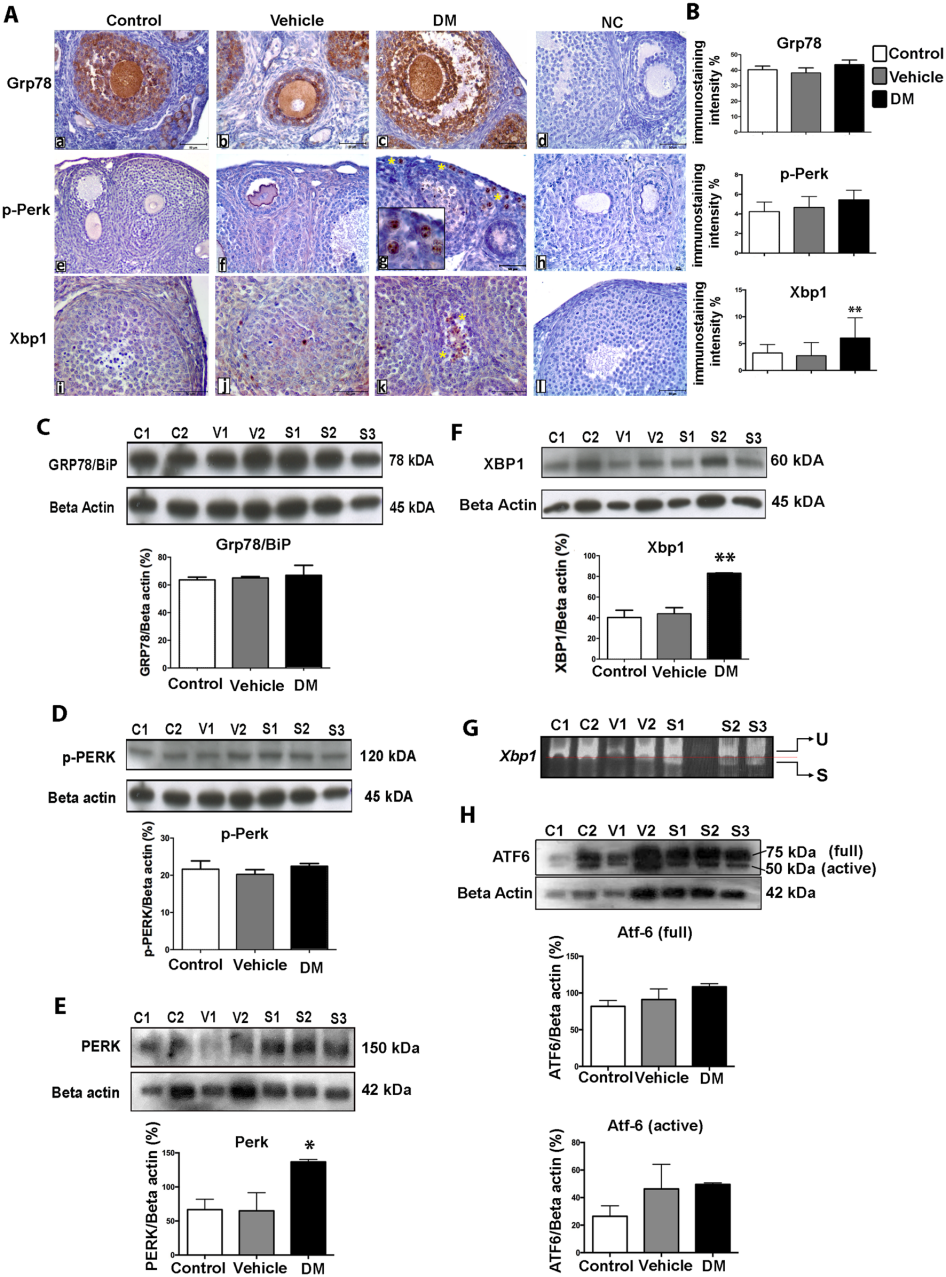
ER stress related IRE1α/XBP1 branch of the UPR is activated in mice ovaries with type 1 DM. **(A)** The expression of Grp78 (a–b–c), p-Perk (e–f–g) and Xbp1 (i–j–k) proteins in control, vehicle and DM group ovaries. Of note, p-Perk is specifically noted only in primordial follicles from DM exposed ovaries (g, zoomed inset). Additionally, accumulated Xbp1 nuclear expression in GCs was noted in the ovaries with DM (yellow asterisks). *NC* negative controls (d–h–l). Scale bars = 50 µm. (**B)** Histograms show immunostaining intensities for Grp78 (top), p-Perk (middle) and Xbps1 (bottom) proteins that were obtained using Image J software. One-way ANOVA followed by TUKEY’s multiple comparisons test, **P = 0.0011 (n = 6 per group). Columns are means ± SD. (**C–F, H)** Total protein quantification of Grp78/BiP **(C)**, p-Perk **(D)**, PERK (total form, **E**), Xbp1 **(F)** and Atf6 (**H**, full and active form) analysed by Western Blotting in ovaries (for the original images for blots please see Supplementary Fig. 1). *C* control (n = 4); *V* vehicle (n = 4); *S* STZ-induced (n = 6). Beta actin is used as an internal control. One-way ANOVA followed by TUKEY’s multiple comparisons test, *P = 0149, **P = 0.0093. Columns are means ± SD. **G.** A representative image of Xbp1 RT-PCR products from ovary groups, *C* control, *V* vehicle, *S* STZ-induced, *U* unspliced Xbp1 (343 bp), *S* spliced Xbp1 (327 bp).

Cells respond to environmentally induced ER stress via UPR, activating downstream pathways that reduce the unfolded protein loading in ER lumen^14,16^. In order to obtain an insight into a potential role of ER stress in ovarian tissue in response to hyperglycaemic environment, we first assessed the activation of the pro-survival UPR signalling. To this end, we analysed the expression profile of Grp78, phosphorylated Perk (p-Perk) and Xbp1 on ovarian tissues by immunohistochemistry. Grp78 was mainly localized in the cytoplasm of granulosa cells and oocytes in the developing follicles of ovaries, with each group showing similar expression levels (Fig. 2A,B). The overall expression intensity of p-Perk, which has a crucial role in inhibiting the initiation of protein translation under ER stress to maintain cellular homeostasis^15^, did not differ between groups (Fig. 2A,B). However, higher p-Perk expression was noted in oocytes within the primordial follicles in mice with DM (Fig. 2A, g-inset). We then assessed another regulator of pro-survival UPR, Xbp1 signalling, which is induced by Atf6 and spliced (Xbp1s) by Ire1α in response to ER stress^26^. We observed a significant increase in of Xbp1 protein expression level, and this was specifically observed in the atretic follicles in mice with DM (Fig. 2A,B). Next, in order to confirm these results from immunohistochemistry, we quantified protein expressions by Western Blotting in whole ovaries. Accordingly, Grp78 showed similar levels between groups (Fig. 2C). Of note, despite undetected difference for p-Perk (Fig. 2D), we found that total Perk was significantly increased in ovaries with DM (Fig. 2E). Validating the immunohistochemistry observations, we found significant upregulation of Xbp1 in ovaries with DM by Western Blotting (Fig. 2F), and further confirmed the splicing of *Xbp1* mRNA in these ovaries (Fig. 2G). Finally, we also analysed the expression of Atf6, the third pro-survival UPR mechanism, which did not show a significant change between groups and only displayed a marginal increase in ovaries with DM (Fig. 2H).

This result suggests that the ER-stress is induced in the ovarian tissue by type 1 DM and, in response, the Ire1/Xbp1 pro-survival signalling is activated via UPR. We next sought to investigate whether this pro-survival signal activation is sufficient to ensure tissue protection/ survival.

### ER-stress induced apoptosis is activated by the Caspase 12 mediated apoptotic pathway in response to hyperglycaemic microenvironment in DM ovaries

Depending on the severity and duration of ER stress, the UPR can initiate pro-apoptotic pathways^14^. After observing the UPR activation through the Ire1/Xbp1 pathway in ovarian tissue with DM, we sought to investigate whether apoptotic UPR signals are induced by assessing markers associated with these pathways, including Chop, Caspase 12 and Cleaved Caspase 3. Chop and Caspase 12 are two independent downstream targets of the UPR, acutely activated in response to permanent ER stress within the cells^27^. Chop is a transcription factor that induces apoptosis by supressing the expression of various pro-survival proteins^19^. Alternatively, activation of Caspase 12 from pro-Caspase 12 is specifically induced by the ER-stress. Caspase 12 triggers a specific cascade concluding with the activation of Caspase 3, a critical executioner of apoptosis^28^.

**Figure 3:**
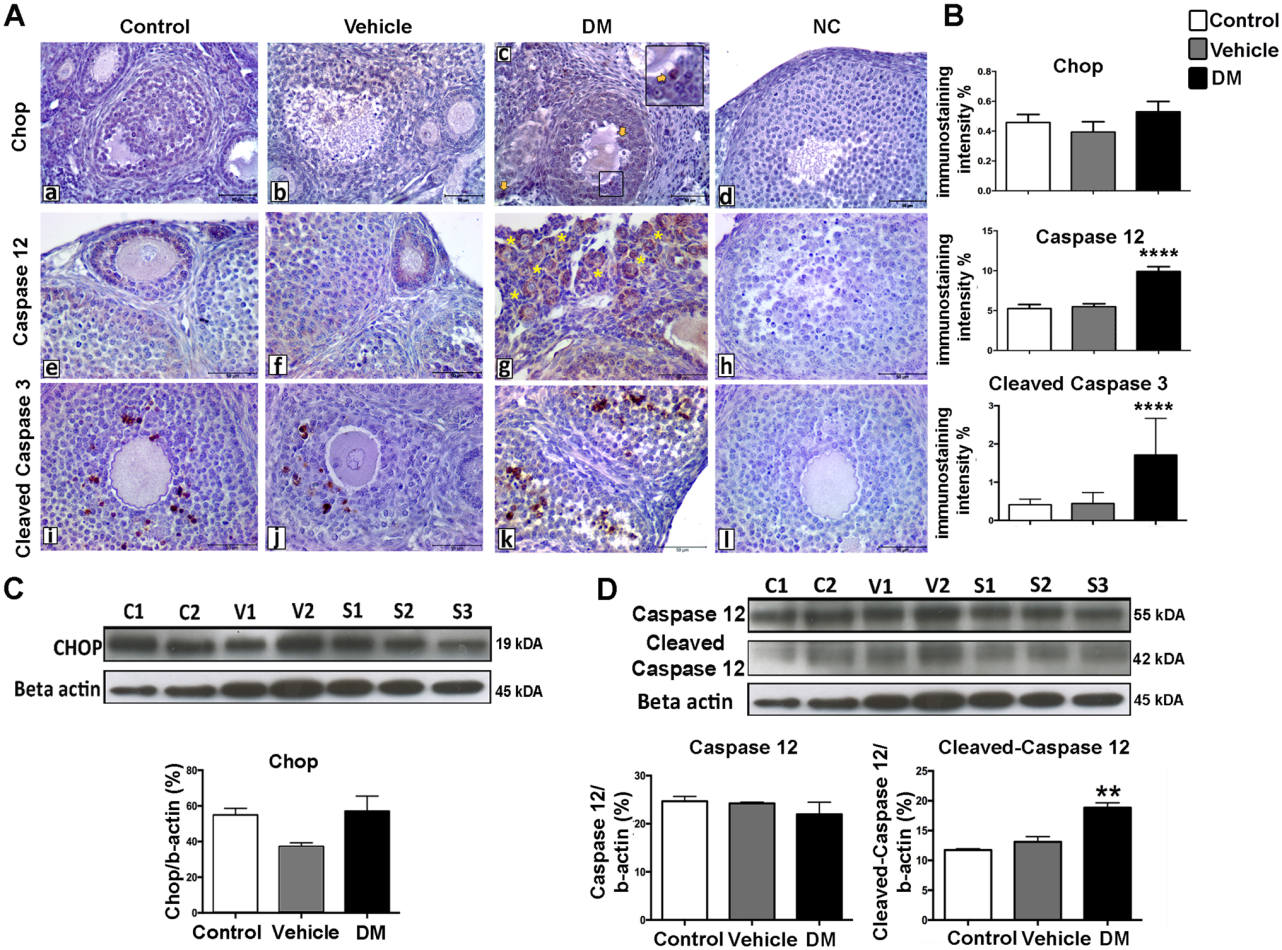
ER stress induced apoptosis is activated by the Caspase 12 mediated intrinsic apoptotic pathway in response to high glucose microenvironment. **(A)** The expression of Chop (a–b–c), Caspase 12 (e–f–g) and Cleaved Caspase 3 (i–j–k) proteins in control, vehicle and DM group ovaries. Caspase 12 (yellow asterisks) and Cleaved Caspase 3 expression was found remarkably increased in ovaries with DM, compared to the control groups. *NC* negative controls (d–h–l). Scale bars = 50 µm. (**B)** Histograms show immunostaining intensities for Chop (top), Caspase 12 (middle) and Cleaved Caspase 3 (bottom) proteins that were obtained using Image J software. One-way ANOVA followed by TUKEY’s multiple comparisons test, ****P < 0.0001 (n = 6 per group). Columns are means ± SD. (**C,D)** Total protein quantification of Chop **(C)**, Caspase 12 and Cleaved Caspase 12 **(D)** analysed by Western Blotting in ovaries (for the original images for blots please see Supplementary Fig. 1). The Cleaved Caspase 12 was normalized to total Caspase 12 expression. *C* control (n = 4), *V* vehicle (n = 4), *S* STZ-induced (n = 6). Beta actin is used as an internal control. One-way ANOVA followed by Tukey’s multiple comparisons test, **P = 0.0089. Columns are means ± SD. The samples derive from the same experiment and that gels/blots were processed in parallel.

Immunohistochemistry analysis showed that while Chop was scarcely expressed in ovaries and did not change between groups (Fig. 3A,B), both Caspase 12 and Cleaved Caspase 3 were significantly increased in granulosa cells of ovaries exposed to DM compared to controls (Fig. 3A,B). We confirmed results from Chop and Caspase 12 (cleaved) with Western Blotting (Fig. 3C,D).

These results indicate that despite the activation of pro-survival UPR through Ire1/Xbp1 pathway, ER stress primarily triggers pro-apoptotic pathways in the ovaries, likely due to the severe disrupted ER homeostasis in the DM condition.

### ER-stress and UPR is activated in early embryos after maternal and in vitro exposure to DM

**Figure 4:**
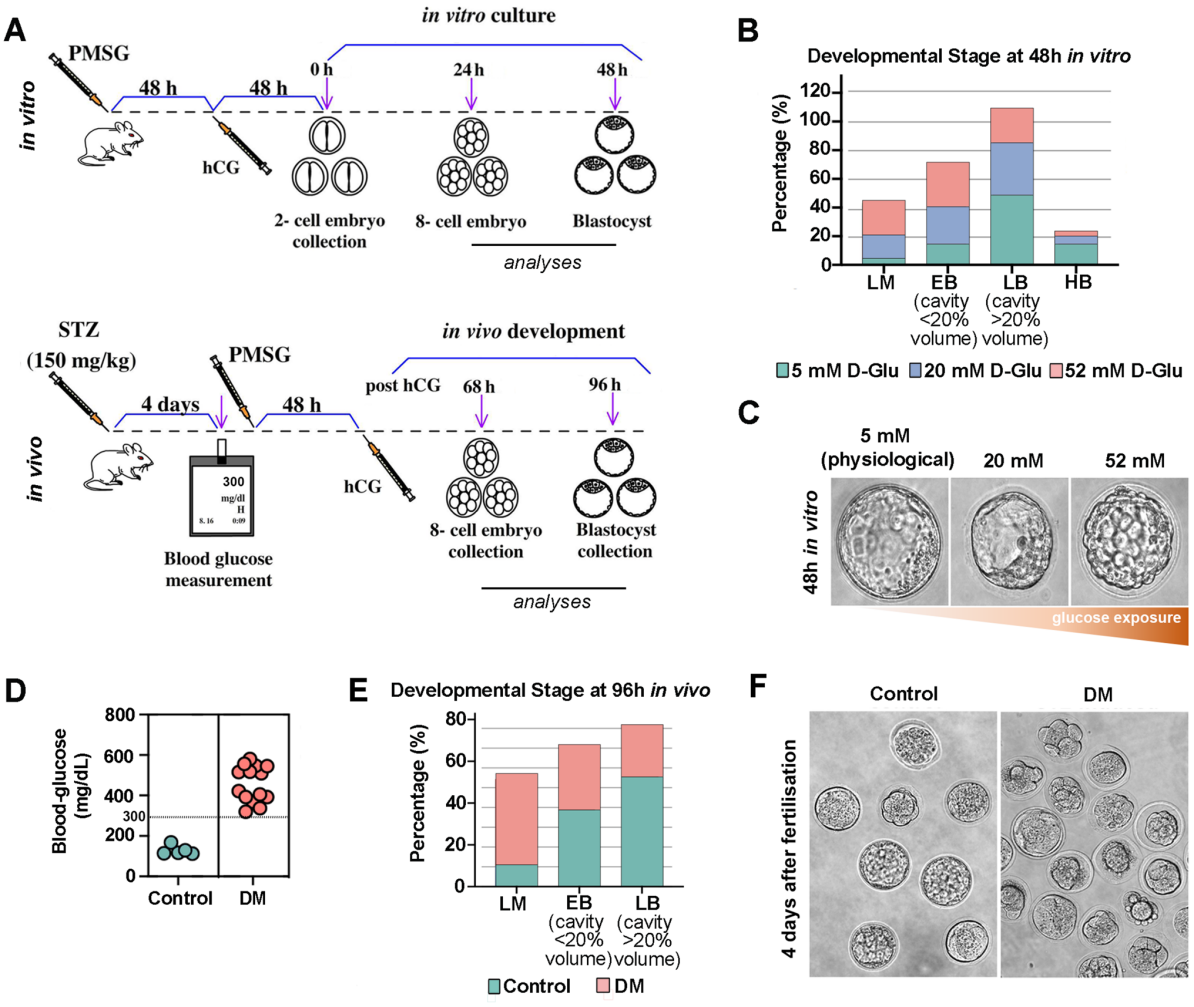
In vivo and in vitro exposure to hyperglycaemia results in delayed pre-implantation embryonic development. **(A)** Schematic shows the experimental approaches applied for in vitro (top) and in vivo (bottom) to model DM microenvironment*,* and following pre-implantation embryo collection. (**B)** Developmental stages observed in pre-implantation embryos following 48 h in vitro culture in 5 mM (physiological control) (n = 76), 20 mM (n = 123) and 52 mM (n = 145) D-Glucose exposed experimental groups. *LM* late morula, *EB* early blastocyst, *LB* late blastocyst, *HB* hatching blastocyst. (**C)** Representative images of blastocyst stage embryos developed in vitro in increasing concentrations of glucose microenvironment. (**D)** Blood glucose level measurement of experimental groups. Mice with glucose level ≥ 300 mg/dL were considered as DM. Control, n = 5; STZ-induced DM, n = 15. (**E)** Developmental stages observed in pre-implantation embryos 96 h after fertilisation in vivo in control (n = 76) and DM (n = 80) experimental groups. *LM* late morula, *EB* early blastocyst, *LB* late blastocyst, *HB* hatching blastocyst. (**F)** Representative images of embryos collected in vivo at 96 h after fertilisation from control and diabetic mothers.

Maternal environment is known to play a central role in dictating developmental outcome in early embryogenesis^1^. To investigate a possible role for ER stress related UPR in pre-implantation embryos in response to environmental DM, we followed two complementary experimental approaches (Fig. 4A). Firstly, to determine in vitro effect of hyperglycaemia on pre-implantation embryo development, we harvested 2-cell stage embryos from control mothers and divided into three groups that received continuous exposure to physiological control (5 mM), or high concentrations of glucose (20 mM and 52 mM) (Fig. 4A). Following in vitro exposure, embryos were analysed for UPR mechanisms after 24 and 48 h in culture (Fig. 4A). In the second experimental design, we collected embryos at embryonic (E) 2.5 or 4 days after fertilisation to analyse morula and blastocyst stage embryos after in vivo exposure to maternal Type 1 DM induced by STZ injection and assessed them for the activation of UPR mechanisms (Fig. 4A).

We first observed that a significant developmental delay in pre-implantation embryos occurs upon exposure to hyperglycaemia both in vitro and in vivo. Two-cell stage embryos cultured for 48 h under hyperglycaemic conditions were delayed with the majority progressing only to the late morula or early blastocyst stage, whereas controls developed to late or hatching blastocysts (Fig. 4B). Furthermore, blastocysts developed under high glucose concentration had enlarged cell size (Fig. 4C). In vivo, after confirming that all mice treated with STZ became diabetic (Fig. 4D), we observed a similar developmental delay in pre-implantation embryos in response to maternal DM (Fig. 4E). Four days after fertilisation, a significant proportion of in vivo developed embryos were observed at morula stage with a lower proportion reaching the late blastocyst stage, compared to those developed in control mice (Fig. 4E,F).

**Figure 5:**
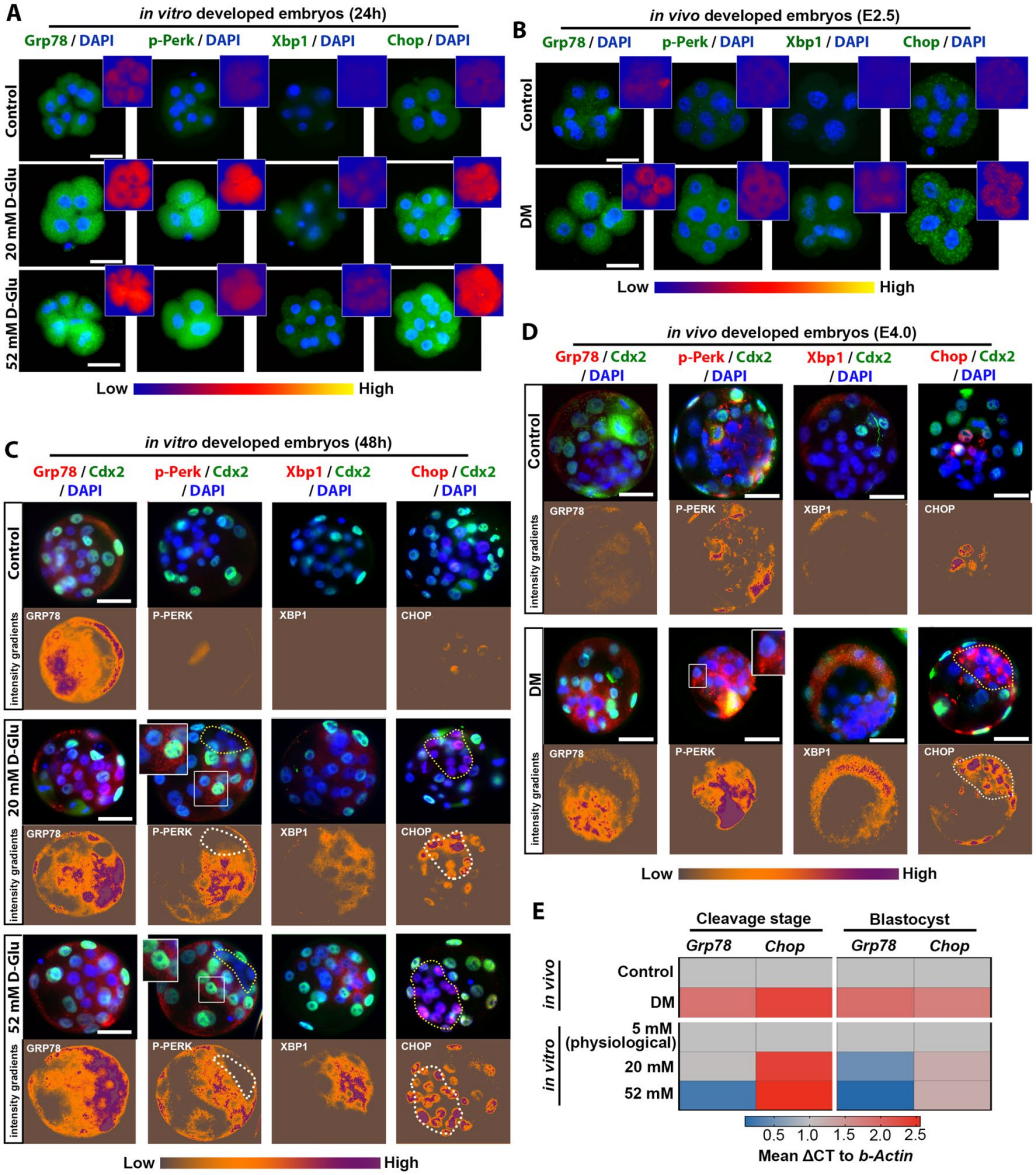
Activation of UPR mechanisms in pre-implantation embryos in response to in vivo and in vitro hyperglycaemic environment. **(A,B)** The expression of Grp78, p-Perk, Xbp1 and Chop proteins in cleavage stage embryos (at 24 h in vitro culture) in 5 mM (physiological control), 20 mM and 52 mM D-Glucose exposed experimental groups **(A)**; and 2.5 days after fertilisation in vivo **(B)**. Green fluorescent, UPR proteins; blue fluorescent, nuclear DAPI labelling. Insets show Grp78, p-Perk, Xbp1 and Chop expression as an intensity gradient map. Scale bars = 50 µm. At least 5 embryos analysed per group. (**C,D)** Double immunofluorescence staining for of Grp78, p-Perk, Chop and Xbp1 in embryos progressed through blastocyst stage in vitro at 48 h **(C)** and in vivo 4 days after fertilisation **(D)**. Red fluorescent, UPR proteins; green fluorescent TE layer marker Cdx2; blue fluorescent, nuclear DAPI labeling. Images below show Grp78, p-Perk, Xbp1 and Chop expression as an intensity gradient map. Yellow and white dashed lines demarcate ICM. Scale bars = 50 µm. At least 5 embryos analysed per group. (**E)** Heat map of *Grp78 and Chop* genes detected by qRT-PCR in embryos from control and diabetic experimental groups (cleavage stage embryos collected at 24 h in vitro or E2.5 in vivo; blastocyst stage embryos collected at 48 h in vitro or E4.0 in vivo). △△Ct quantification is performed (numbers represent fold change difference). n = 30 embryos pooled over 3 independent experiments per sample. *Beta Actin* used as internal control. Blue and red indicate downregulation and upregulation, respectively.

**Figure 6:**
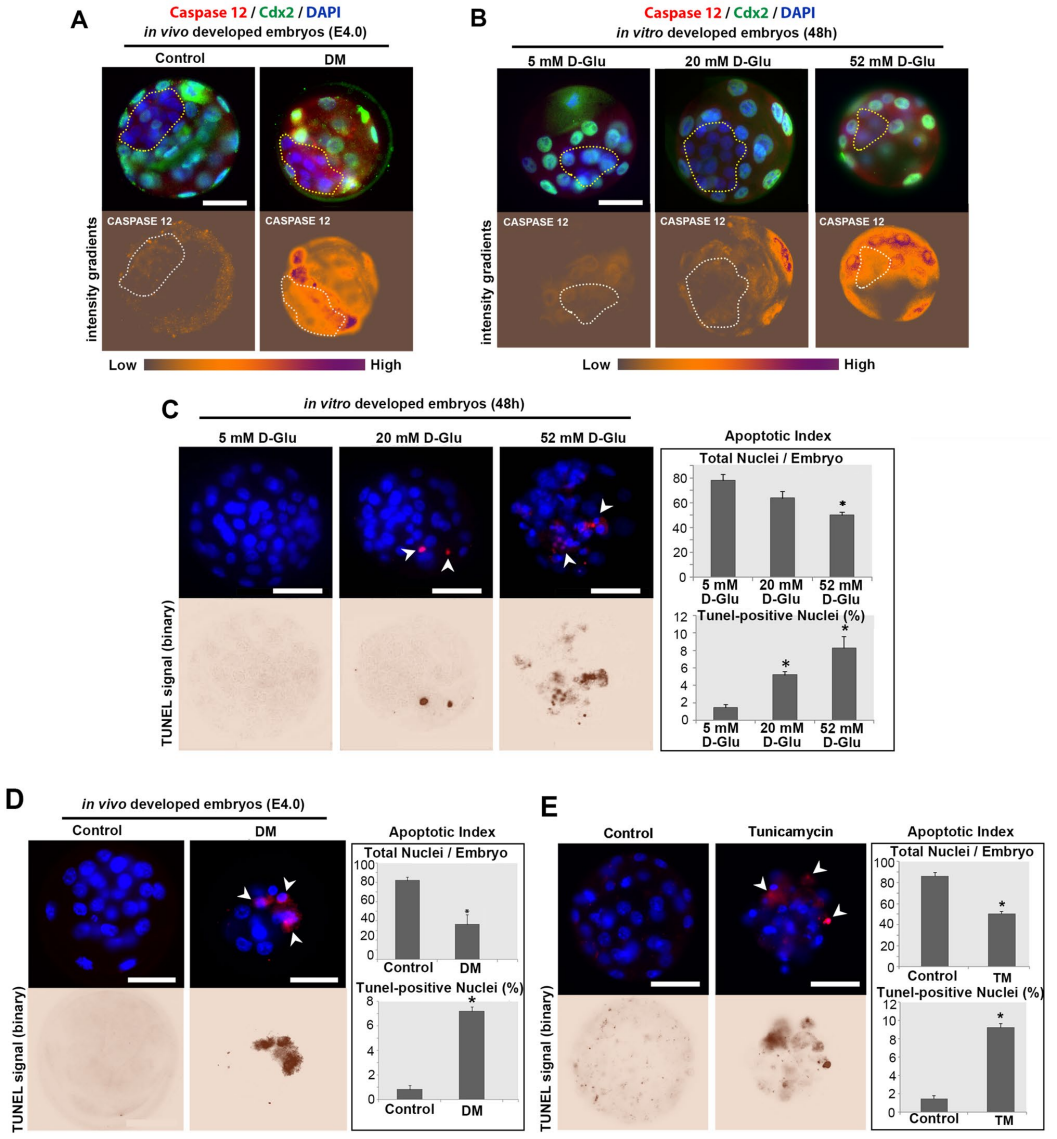
Cell death assessment in blastocysts developed in hyperglycaemic conditions in vitro and in vivo. **(A,B)** The expression of Caspase 12 protein at 4 days after fertilisation in vivo **(A)**; and in blastocyst stage embryos (at 48 h in vitro culture) in 5 mM (physiological), 20 mM and 52 mM D-Glucose exposed experimental groups **(B)**. Red fluorescent, Caspase 12; green fluorescent TE layer marker Cdx2; blue fluorescent, nuclear DAPI labelling. Images below show Caspase 12 expression as an intensity gradient map. Yellow and white dashed lines demarcate ICM. Scale bars = 50 µm. At least 5 embryos analysed per group. (**C,D)** Cell death rates and nuclear cell counts were determined in the blastocysts exposed to hyperglycaemic conditions in vitro **(B)** and in vivo **(C)** by the TUNEL assay. Red fluorescent, TUNEL marking that binds to DNA fractures; blue shows the DAPI staining bound to fluorescent nuclear content. White arrows show TUNEL-positive nuclear fragments; binary images below highlight TUNEL signal. Scale bars = 50 µm. One-way ANOVA followed by TUKEY’s multiple comparisons test, *P < *0.05*. n = 10 per group. All experiments were performed in triplicate. Columns are means ± SD. (**E)** Cell death rates and nuclear cell counts in control (n = 10) and Tunicamycin (TM)-treated (n = 5) embryos. Red fluorescent, TUNEL marking that binds to DNA fractures; blue shows the DAPI staining bound to fluorescent nuclear content. White arrows show TUNEL-positive nuclear fragments; binary images below highlight TUNEL signal. Student’s t test, *P < *0.05*. n = 10 per group. Columns are means ± SD.

In order to gain insight into causal mechanisms of this developmental delay in response to diabetic microenvironment, we next analysed the activation of ER stress and UPR in the early embryos developed in vitro and in vivo. We found Grp78 and p-Perk were highly expressed in cleavage stage embryos in response to hyperglycaemia both in vitro and in vivo (Fig. 5A,B). This suggests that in response to increased ER stress in early embryos by hyperglycaemia, the pro-survival UPR signalling is activated (Fig. 5A,B). We also found that Xbp1s, another pro-survival regulator of UPR that functions to alleviate intracellular ER stress, was not expressed in these embryos (Fig. 5A,B). In blastocyst stage embryos, we found that the Grp78 expression was higher after in vivo DM exposure, and both pro-survival UPR markers p-Perk and Xbp1 were increased in response to hyperglycaemia in vitro and in vivo (Fig. 5C,D). Of note, p-Perk expression was exclusively observed in trophectoderm (TE) lineage, but not in inner cell mass (ICM) (Fig. 5C, insets). Conversely, we observed higher pro-apoptotic Chop expression in ICM than TE lineage (Fig. 5C,D). This indicated a possible lineage specific divergent UPR signalling activation in blastocyst stage embryos. Although high expression of Chop protein was also found in hyperglycaemia-exposed cleavage stage embryos, the enrichment was cytosolic rather than nuclear (Fig. 5A,B). The accumulation of Chop in the nucleus at blastocyst stage indicated its canonical activation and upregulation of apoptotic signalling pathways in response to hyperglycaemia (Fig. 5C,D). This increase in *Grp78* and *Chop* expression was also confirmed by quantitative RT-PCR (qRT-PCR) in embryos (Fig. 5E). Additionally, we analysed blastocyst stage embryos for Caspase 12 activation after in vivo and in vitro exposure to DM. We found that in vivo maternal exposure and in vitro hyperglycaemia at the highest concentration resulted in increase in Caspase 12 expression, enriched mostly in nuclei of the TE lineage (Fig. 6A,B).

We finally confirmed cell death by analysing apoptotic nuclear fragmentation with TUNEL assay^30^ in blastocysts developed in vivo or in vitro under the hyperglycaemic environment. Following 48 h of in vitro high glucose exposure, blastocysts contained fewer cells and the percentage of apoptotic nuclear fragmentation was significantly increased (Fig. 6C). Similarly, embryos from diabetic mothers at 4 days after fertilisation demonstrated the disrupted in vivo development, containing severely reduced number of cells and a significant increase in apoptotic nuclear fragmentation (Fig. 6D). To confirm these results we used Tunicamycin (TM) as a positive control, known to induce UPR by blocking the synthesis of N-linked glycoproteins (N-glycans)^29^. We observed similar outcomes in response to TM treatment which promoted nuclear fragmentation and increased cell death (Fig. 6E), affecting the blastocyst formation in vitro as shown previously^30^.

Altogether these results revealed that ER stress is induced in pre-implantation embryos in response to maternal DM, resulting in delayed development and activation of apoptotic signalling.

## Discussion

Women of reproductive age who are affected by DM, a state of chronic hyperglycaemia, suffer from low fertility, the risk of spontaneous abortions, and having infants with major congenital malformations^22,31^. Although this epidemiology has been well charted, the aetiology of these events still largely remains unclear. Previous studies have reported that ER stress is involved in the pathogenesis of metabolic disorders, including peripheral insulin resistance and type 2 diabetes^20,21^. Thus intervention of ER stress may offer new opportunities for treating diabetes. In the present study, in order to obtain a better understanding of the pathophysiology of type 1 DM on ovarian function and early embryo development, we analysed the activation of UPR, an autonomous adaptive response to intracellular ER stress.

The ER controls multiple distinct cellular functions including processes essential for cell growth and survival^13,14^. Dysregulation of protein synthesis and trafficking is manifested as ER-stress, presenting a challenge for both the cell itself as well as its neighbours via non-autonomous effects^14,32^. UPR, a self-protective and adaptive mechanism, is activated in response to ER-stress, with divergent cellular outcomes depending on severity^13,32^. If cells cannot restore ER homeostasis and cannot resolve the burden of aggregated unfolded proteins properly, UPR mechanisms are activated to eliminate damaged cells by apoptosis^13,14^.

We first analysed the activation of ER stress-related UPR transducers in ovarian tissue in response to 2-weeks of DM in mice of reproductive age (approximately after 3 successive ovarian cycle, at pubertal age between 5–7 weeks). In mammalians, the activation of the UPR is mediated by three distinct ER stress sensors: PERK, ATF6, and IRE1α/XBP1. We found no difference in expression levels of p-Perk protein between groups, despite a significant increase in total PERK protein levels in ovaries with DM. Since the most immediate response to ER stress is known to be initiated by the PERK^33^, increased levels of total PERK may suggest that this ER stress sensor could have contributed to the activation of the UPR. However, after observing no significant change in the levels of phosphorylated PERK (p-PERK) we conclude that the Grp78/p-Perk pro-survival pathway is not involved in the ER stress response under diabetic conditions in ovaries. Similarly, we did not find any significant difference for ATF6 between groups. Our analyses did however show significantly elevated levels of Xbp1 in diabetic ovaries. This suggests that UPR is mediated though the pro-survival transducer IRE1α/XBP1 in response to ER stress in diabetic ovaries. As shown before, IRE1α/XBP1 induces expression of genes encoding ER protein chaperones and endoplasmic-reticulum-associated protein degradation (ERAD) pathways that function in cell survival^34^. Thus, it is likely that the activation of Xbp1 in ovarian tissue acts to relieve the ER stress via ERAD pathway in response to DM. Despite this result showing activation of pro-survival signalling in ovarian tissue, we still observe tissue malformation and evidence of cell death in the ovarian tissue. Indeed, at the molecular level we further found significant upregulation of both Caspase 12 and Cleaved-Caspase 3 in ovaries with DM. We thus suggest that although an adaptive pro-survival response is initiated by IRE1α/XBP1 transducers, it is likely that the excessive ER stress caused by type 1 DM favours an apoptotic mechanism through the caspase-12–9–3 signalling cascade in ovarian tissue. Of note, the other pro-apoptotic transducer of ER stress-mediated UPR, Chop, was not upregulated in ovaries with DM. This indicates that a selective activation of cell death occurs in type 1 DM ovarian tissue via ER-associated Caspase 12, and not the Chop-signalling pathway.

Diabetic pregnancies are associated with an increased risk for congenital malformations in the offspring^31^. In our experimental model, pre-implantation embryos become highly sensitive to maternal hyperglycaemia and exhibited developmental delay, as previously reported^23^. Interestingly, we found that multiple ER stress transducers were activated by UPR mechanisms in pre-implantation embryos. We observed that while cleavage stage embryos activate Grp78/p-Perk pathway, and not the IRE1α/XBP1 pathway, in response to both in vivo and in vitro hyperglycaemia; at the blastocyst stage both pro-survival UPR transducers were found to be activated. Interestingly, we observed differential activation for UPR and stress-response pathways in early embryo lineages. We observed exclusive expression of p-Perk in the TE lineage, whereas there was higher Chop expression in the ICM. This implies a heterogeneity in causation of malformations observed in placental and foetal development later in pregnancy. This result is in line with previous reports that link the PERK branch of UPR signalling in ER stress with preeclampsia, a severe placenta-related pregnancy complication^35,36^. Furthermore, we found elevated Caspase 12 expression in the TE lineage in response to DM in both in vivo and in vitro developed blastocysts. It is likely that the severity of ER stress and UPR determine trophoblast cell fate later in placental formation and function. It may be that at low levels, UPR functions as a protective mechanism safeguarding tissue development by the activation of PERK pathway, whereas at high levels UPR induces cell death by activation of the Caspase 12 pathway.

Interestingly, the ICM responded differently to ER stress by the accumulation of pro-apoptotic Chop within the ICM under severe environmental stress. Thus, embryonic and placental precursor may manage ER-stress through distinct mechanisms: while Chop signalling is activated within ICM, Caspase 12 signalling is triggered within TE to induce pro-apoptotic arm of UPR in response to hyperglycaemic environmental stress.

In summary, our study demonstrates that type 1 DM causes excessive ER stress and/or insufficient adaptive UPR to ER stress in ovarian tissue and early pre-implantation embryogenesis in mouse. Despite that the pro-survival UPR mechanism is activated, it is opposed by a higher degree of apoptotic signalling ultimately causing widespread cell death. We speculate that the activation of pro-survival pathways may play role in preventing a complete cell death resulting in female sterility and/or abortion of the embryos. These results may help to explain previously reported characteristic malformations in ovarian function and post-implantation embryogenesis under diabetic conditions, which may become apparent due to excreted cell death factors as shown in this study. Thus, in the light of our results and previous studies, we suggest that the altered cellular physiology and function caused by ER stress in response to maternal DM can lead to adaptive development in surviving embryos that are likely to manifest as malformations in offspring. This study may lead to new approaches to understanding the mechanism of adverse effects due to diabetes in the ovary and in the discovery of new treatments.

## Materials and methods

### Animals

All animal experiments were carried out in compliance with the National Institutes of Health guide for the care and use of laboratory animals (NIH Publications) as well as the ARRIVE guidelines and approved by the Akdeniz University Institutional Animal Care and Use Committee (Protocol No. 2014.05.02). Mice were maintained in accordance with national and international guidelines. All mice were caged in a standard lighting (12-h light–12-h darkness shifting cycle), temperature (21 ± 2 °C) and humidity (60 ± 10%) controlled room with access to water and food ad libitum.

### STZ model of diabetes mellitus

Diabetes was induced by a single intraperitoneal injection of STZ (Sigma Aldrich) to 5 weeks old BALB/c female mice according to published methods^24,25^. Briefly, the STZ compound was dissolved in a fresh citrate buffer (0.1 M citrate, pH 4.5), and injected intraperitoneally (90 mg/kg/day) within 15 min of dissolution. The vehicle group were treated with citrate buffer only and the control group received no treatment. After two weeks of injection, the tail venous blood sampling from individuals was collected. Animals with glucose level ≥ 300 mg/dL were considered as STZ-induced diabetic mice^43^.

### Ovarian tissue collection and processing

Following 14 days of STZ injection, female mice from each experimental group was sacrificed and ovaries were collected under sterile conditions. For each female, left ovary was used for paraffin embedding and right ovary was used for Western blotting. For immunohistochemistry, all ovarian tissue samples were fixed at + 4 °C for 6 h in in Bouin’s fixative (75% of saturated picric acid [Sigma-Aldrich], 25% of formalin [Merck], 5% of glacial acetic acid [Sigma-Aldrich]). Tissue samples were then transferred through series of ethanol baths of increasing concentrations, subsequently cleared with xylene and embedded in paraffin (Sigma) blocks. For Western blotting, ovarian tissue samples were stored in liquid nitrogen (− 196 °C) until required.

### Ovarian histomorphology and atretic follicle counting

Serial sections with 5 µm thickness was stained with Harris Hematoxylin Eosin and examined for ovarian histomorphology and follicle counting under light microscope (Olympus BX53). The total number of healthy and atretic follicle counting was performed in one of every five sections of the ovarian serial sections of mice from each group. Follicles that contain oocytes with irregular plasma membranes and/or oocytes are eosinophilic and/or granulosa cells with pyknotically condensed nuclei are considered atretic. Healthy follicles were counted according to the Pederson’s classification^37^.

### Immunohistochemistry on tissue sections and quantitative analysis of immunohistological staining

Serial sections at 5 μm thickness were cut from paraffin embedded ovarian blocks using a rotary microtome (Leica). Sections first were deparaffinised in xylene, then rehydrated through a graded series of ethanol. Subsequently, the sections were boiled in citrate buffer (pH: 6.0) for 10 min for epitope retrieval and cooled down for 20 min at room temperature. In order to block endogenous peroxidase, %3 hydrogen peroxide in methanol solution were used for 15 min at room temperature. Following a blocking step with UltraV block (Thermo Fisher) for 7 min at room temperature, the slides were incubated with primary antibodies overnight at 4 °C in a humidified chamber. For negative controls, primary antibodies were replaced with normal rabbit serum at the same concentration. Following day, the slides were wasted with PBS three times, and incubated with secondary antibodies 1 h at room temperature (1:400). After streptavidin peroxidase complex (Invitrogen) incubation for 20 min, diaminobenzidine (DAB) chromogen (Sigma) was applied according to the manufacturer's instructions (Thermo Fisher). For nuclear counterstaining, Mayer's haematoxylin (Merck) was used. After permanent mounting, slides were analysed a bright-field microscope (Zeiss). The antibodies and working concentrations used are given in Supplementary Table 1. In order to quantify the immunohistological staining for each protein, TIFF images were imported to ImageJ/Fiji software and threshold function applied in order to separate the signal from the background and the mean signal intensity was measured by the “measure” function^38–41^. The mean intensity of the background was obtained by averaging the values of negative control images that had been treated with secondary antibody only. The staining intensity level value was calculated by dividing the mean signal intensity above the background for a minimum 20 images per mouse for a minimum 3 mice per experimental group.

### Protein extraction and Western blotting

Ovarian tissue samples obtained from control and experimental groups were put into tubes with ceramic beads (Magna Lyser Green Beads, Roche). After adding 300 µl Lysis buffer and 5 µl protease inhibitor cocktail in each tube, samples were centrifuged at 6500 rpm for 45 s with a Magna Lyser instrument (Roche). Supernatants from each sample were collected and stored at - 20 °C until required.

Ovarian tissue lysates were separated by SDS-PAGE in 7.5% (for PERK, GRP78 and XBP-1) and in 15% (for Chop and Caspase 12). After electrophoresis, proteins were transferred onto polyvinylidene difluoride (PVDF) membranes (Biorad). To reduce the nonspecific binding, membranes were blocked with 5% non-fat dry milk in TBS-T buffer (0.1% Tween-20 in Tris-buffered saline) for 45 min. Membranes were then incubated with primary antibodies overnight at + 4 °C. Following day, membranes were first washed with TBS-T three times for 10 min each, and then incubated with peroxidase-labeled goat anti-rabbit IgG (Vector) for 1 h at room temperature. For visualisation, chemiluminescence base SuperSignal CL-HRP Substrate System (Thermo Scientific) were used according to manufacturer’s instructions, and subsequently exposured to the Hyperfilm (Amersham) in dark. β-actin (Cell Signaling Technology) was used for internal control. The antibodies and working concentrations used are given in Supplementary Table 1. When membrane stripping is performed between antibody probing, the blot is incubated in stripping buffer (200 mM glycine, 3.5 mM SDS, 1% (v/v) Tween 20, pH 2.2) for 10 min room temperature. Following PBS washing for 20 min and TBS-T buffer for 10 min, the blot is blocked for an hour in 5% (w/v) non-fat dried milk in TBS-T.

### Superovulation and embryo recovery

Superovulation is performed with intraperitoneal injection of 5 IU of pregnant mare's serum gonadotropin (PMSG, Sigma-Aldrich) and 5 IU of human chorionic gonadotrophin (hCG, Sigma-Aldrich) 48 h later, and crossed with 12-week-old BALB/c males. The time when a vaginal plug is observed, considered as 0.5-day post coitum (E0.5). Cleavage stage embryos were obtained by puncturing the oviduct, blastocysts were recovered by uterine flushing. For in vitro experiments, embryos were cultured in KSOM/0.25% BSA (v/v) (Millipore) medium under mineral oil (Sigma) in 5% CO_2_ at 37.5 °C.

### Whole mount double immunofluorescence

Fixation is performed with 4% PFA for 20 min at room temperature. For washing, PBS-T [phosphate-buffered saline (PBS) containing 0.05% Tween-20] solution is used. For cellular permeabilization, 0.3% Triton-X-100, 0.1% glycine solution used for 30 min at + 4 °C. Incubation with mixture of primary antibodies was performed overnight at + 4 °C, in blocking buffer containing 10% FBS in PBS-T. Incubation with secondary antibodies was performed for 1 h at room temperature, in blocking buffer containing 10% FBS in PBS-T. For DNA labelling, DAPI (1 mg/ml) used for for 10 min at room temperature (Sigma-Aldrich). Embryos were then mounted onto glass slides in a glycerol:PBS (1:1) mixture and finally analysed with a florescence microscope (Olympus). The antibodies and working concentrations used are given in Supplementary Table 1.

### TUNEL assay

The original source of method description for TUNEL assay protocol can be found in our previous study^42^. Fixation is performed with 4% PFA for 20 min at room temperature. TUNEL labelling is performed according to the manufacturer’s instructions (Roche). For DNA labelling, DAPI (1 mg/ml) used for 10 min at room temperature (Sigma-Aldrich). Embryos were then mounted onto glass slides in a glycerol:PBS (1:1) mixture and finally analysed with a florescence microscope (Olympus). Each embryo was analysed for total number of nuclei and number of TUNEL-labelled nuclei.

### RNA extraction and reverse transcription

The original source of method description for RNA extraction and reverse transcription protocol can be found in our previous study^42^. A pool of 30 pre-implantation embryos per group was collected and stored in lysis buffer at − 80 °C until required. Total RNA was extracted using the RNAqueous-Micro Kit (Ambion) according to the manufacturer’s instructions. Then, the Reverse Transcription reaction was performed using the RETROscript kit (Ambion) according to manufacturer’s instructions.

### PCR amplification

The original source of method description for PCR amplification protocol can be found in our previous study^42^. The qRT-PCR reactions were performed with the SYBR green supermix (Qiagen) and a Rotor Gene (Corbett Research). The qRT-PCR primers used are given in Supplementary Table 2. The relative gene expression levels were assessed by the 2^−ΔΔCt^ (cycle threshold) method, with Beta Actin as an endogenous control. Xbp1 PCR was done as described previously^43^. PCR amplification with Taq DNA polymerase (GeneDirex) was performed with the cycling conditions of 94 °C for 5 min, followed by 40 cycles of 94 °C for 30 s, 60 °C for 30 s, and 72 °C for 30 s. PCR products were separated by electrophoresis on a 1.2% agarose gel (Invitrogen) and visualized under UV light.

### Statistical analysis

For immunohistochemistry and western blot analysis, the intensity analysis and quantifications of protein expression levels were evaluated by Image J software. Comparisons tests for experimental are indicated in each figure legends. All statistical calculations were performed using Graphpad Prism 6.0 software (San Diego, CA). Differences were considered significant at *P* < 0.05. All error bars represent standard deviation (± SD) unless otherwise is noted in figure legends.

## Supplementary Information

Supplementary Information 1.
