# Supplementary material for: Unfolded protein response triggers differential apoptotic mechanisms in ovaries and early embryos exposed to maternal type 1 diabetes
Source: Sci Rep. 2021 Jun 17;11:12759. doi: 10.1038/s41598-021-92093-3 (PMC8211688; doi:10.1038/s41598-021-92093-3)
Supplement: Supplementary file 1 — Supplementary Information 1. [file 41598_2021_92093_MOESM1_ESM.pdf]

# Unfolded Protein Response Triggers Differential Apoptotic Mechanisms in Ovaries and Early Embryos Exposed to Maternal Type 1 Diabetes

**Aslı Okan[1], Necdet Demir[1*] and Berna Sozen[2*]**

[1]Department of Histology and Embryology, School of Medicine, Akdeniz University, Antalya, Turkey 07070
[2]Department of Genetics, Yale School of Medicine, Yale University, New Haven, CT, USA 06520

***Correspondence to**
**Berna Sozen, PhD**
Yale University
Department of Genetics, School of Medicine,
New Haven, Connecticut 06510, USA
Phone: +1-213-249-3796
Email: berna.sozen@yale.edu

**Necdet Demir, PhD**
Akdeniz University,
Faculty of Medicine,
Department of Histology and Embryology,
Campus, 07070, Antalya/ TURKEY
Phone: + (90) 242 249 68 84
Email: ndemir@akdeniz.edu.tr

# Supplementary Figure 1-

The original unprocessed images for Western blot and the target panels were marked in red boxes.

**Figure 2C**

**Grp78/BiP**

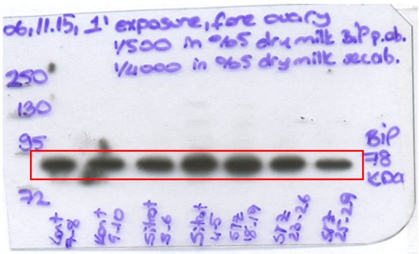


**Beta actin**

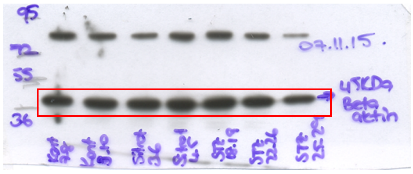


**Figure 2D**

**p-Perk**

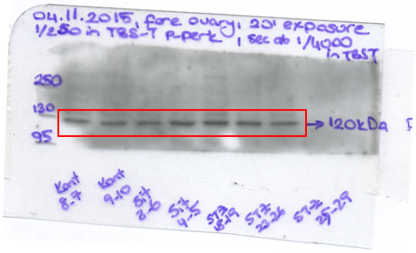


**Beta actin**

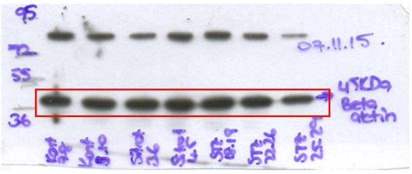


**Figure 2E**

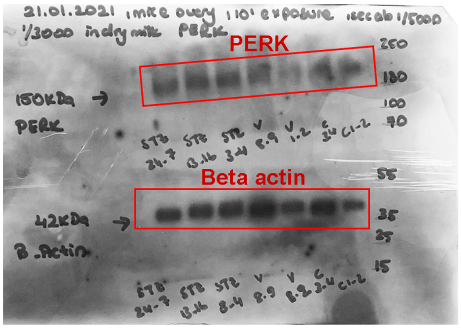


**Figure 2F**

**Xbp1**

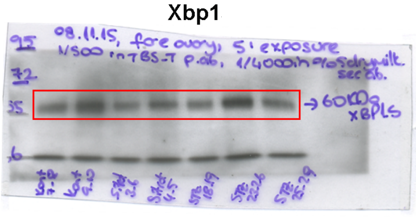


**Beta actin**

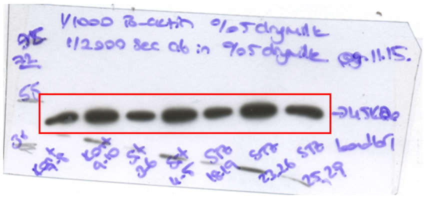


**Figure 2H**

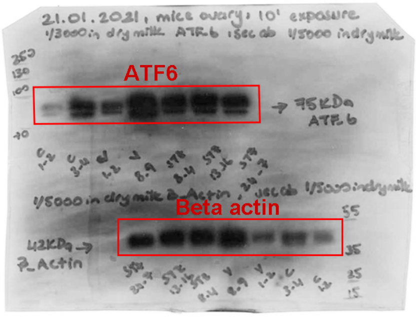


**Figure 3C**

**Chop**

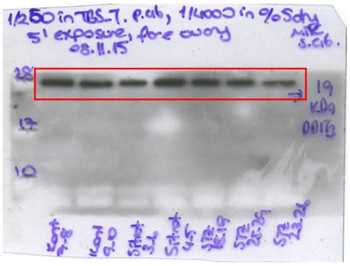


**Beta actin**

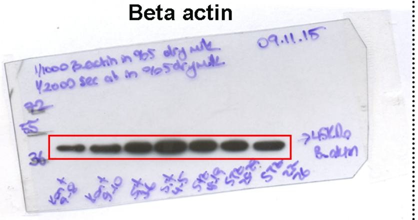


**Figure 3D**

**Caspase12/Cleaved Caspase12**

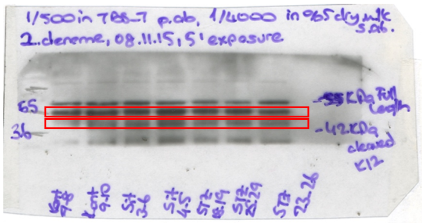


**Beta actin**

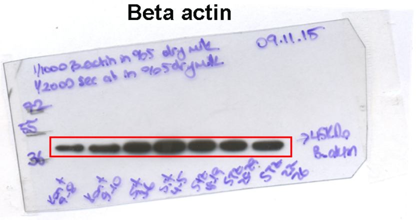

# Supplementary table 1- Antibodies used in this study

| Antibody (species) | Vendor | Number | Dilution |
|---|---|---|---|
| **GRP78/BiP** | Cell Signaling Technology | #3177 | IHC-P 1:600<br>IF 1:100<br>WB 1:500 |
| **P-PERK** | Bioss Antibodies | BS-3330R | IHC-P 1:75<br>IF 1:50<br>WB 1:250 |
| **Caspase 12** | Cell Signaling Technology | #2202S | IHC-P 1:100<br>IF 1:50<br>WB 1:500 |
| **Cleaved Caspase 3** | Cell Signaling Technology | 9661S | IHC-P 1:100 |
| **CHOP/DDIT3** | Abcam | ab179823 | IHC-P 1:50<br>IF 1:50<br>WB 1:250 |
| **XBP1** | Bioss Antibodies | BS-1668R | IHC-P 1:100<br>IF 1:50<br>WB 1:500 |
| **Beta Actin** | Cell Signaling Technology | #4970 | WB 1:1000 |
| **ATF6** | Abcam | ab203119 | WB 1:3000 |
| **PERK (total)** | Abcam | ab229912 | WB 1:3000 |

# Supplementary table 2- qPCR primers used in this study

| Gene | Forward (5' to 3') | Reverse (5' to 3') |
|---|---|---|
| ***Grp78/Bip*** | ‘GAGGTGGGCAAACCAAGACATT’ | ‘TCGCTGGGCATCATTGAAGTAAG’ |
| ***Ddit3/Chop*** | ‘TCTTGACCCTGCGTCCCTAG’ | ‘TGGGCACTGACCACTCTGTTT’ |
| ***B-Actin*** | ‘TGCGTGACATCAAAGAGAAG’ | ‘CGGATGTCAACGTCACACTT’ |
| ***Xbp1*** | ‘GAACACGCTTGGGAATGGACAC’ | ‘AGAAAGGGAGGCTGGTAAGGAAC’ |
